# Risk Factors of Marginal Cord Insertion in Singleton Pregnancies: A Systematic Review and Meta-Analysis

**DOI:** 10.3390/jcm13237438

**Published:** 2024-12-06

**Authors:** Antonios Siargkas, Ioannis Tsakiridis, Athanasios Gatsis, Catalina De Paco Matallana, Maria Mar Gil, Petya Chaveeva, Themistoklis Dagklis

**Affiliations:** 1 Third Department of Obstetrics and Gynecology, Faculty of Health Sciences, School of Medicine, Aristotle University of Thessaloniki, Agiou Dimitriou, 54124 Thessaloniki, Greece; antonis.siargkas@gmail.com (A.S.);; 2 Institute for Biomedical Research of Murcia, IMIB-Arrixaca, El Palmar, Faculty of Medicine, Universidad de Murcia, 30120 Murcia, Spain; 3 Maternal Fetal Medicine Unit, Department Obstetrics and Gynecology, Virgen de la Arrixaca, 30120 Murcia, Spain; 4 School of Medicine, Universidad Francisco de Vitoria, 28223 Madrid, Spain; 5 Department of Obstetrics and Gynecology, Hospital Universitario de Torrejón, 28850 Madrid, Spain; 6 Ultrasound and Fetal Medicine Unit, Obstetrics and Gynecology Department, Hospital Universitario La Paz, 28046 Madrid, Spain; 7 Department of Obstetrics and Gynecology, Faculty of Medicine, Medical University of Pleven, 5800 Pleven, Bulgaria; 8 Fetal Medicine Unit, Dr. Shterev Hospital, 1330 Sofia, Bulgaria

**Keywords:** abnormal cord insertion, peripheral cord insertion, assisted reproductive technologies, chronic hypertension, placenta previa, nulliparity, Cesarean section, risk factors

## Abstract

**Background:** Marginal cord insertion (MCI) is increasingly recognized as a pathological variation that necessitates early diagnosis. Identifying the risk factors associated with MCI is essential for improving prenatal screening and optimizing management strategies. Our meta-analysis systematically and quantitatively synthesizes the current evidence on various potential risk factors for MCI. **Methods:** This systematic review and meta-analysis adhered to the PRISMA and MOOSE guidelines. Comprehensive searches were performed in three databases up until 6 May 2024, identifying observational cohort and case–control studies that examined risk factors for MCI in singleton pregnancies compared with central or eccentric cord insertion. Quality and risk of bias assessment were performed using the Newcastle–Ottawa Scale and the Quality In Prognosis Studies tool, respectively. Statistical analyses employed random-effects models to calculate relative risks (RR) and mean differences with their 95% confidence intervals (95% CI). Heterogeneity was assessed via Cochran’s Q and I^2^ statistics. **Results:** A total of 18 studies (14 cohort and 4 case–control), encompassing 51,463 MCI cases and 901,020 control cases, were included. The meta-analysis revealed a prevalence of MCI at 5.71% among singleton pregnancies. Significant risk factors for MCI included the use of assisted reproductive technology (RR = 1.55; 95% CI: 1.34–1.78), chronic hypertension (RR = 1.47; 95% CI: 1.11–1.95), placenta previa (RR = 1.83; 95% CI: 1.62–2.08), and nulliparity (RR = 1.18; 95% CI: 1.08–1.30). No significant associations were found for smoking, maternal age, prior Cesarean section, preexisting diabetes, or Caucasian ethnicity. Sensitivity analyses corroborated the robustness of these findings. **Conclusions:** This meta-analysis identified assisted reproductive technology, chronic hypertension, placenta previa, and nulliparity as significant risk factors for marginal cord insertion in singleton pregnancies. These findings can inform the development of prenatal screening protocols and enable targeted screenings for high-risk populations.

## 1. Introduction

Umbilical cord insertion (UCI) typically takes place near the central part of the placenta, commonly referred to as central or eccentric cord insertion [1,2]. Variants of UCI include marginal cord insertion (MCI) and velamentous cord insertion (VCI), with VCI being the most extreme deviation, in which the UCI is located outside the placental margin [3,4]; VCI has been associated with various adverse pregnancy outcomes [5]. MCI, where the cord attaches within the placental boundaries but close to the edge (within 2 to 3 cm of the margin), is observed in about 6.15% of singleton pregnancies [6].

Although individual studies have struggled to reach consensus on the associations between MCI and adverse pregnancy outcomes, a recent meta-analysis incorporating data from 15 studies has yielded significant findings [6]. The association of MCI with adverse pregnancy outcomes could be explained by the impact of non-central UCI on placental efficiency and the less dense distribution of chorionic vessels [7]. These insights, combined with advancements in ultrasonography over recent decades, have enabled prenatal visualization of UCI and emphasize the need for a more systematic diagnostic approach [8]. Despite this, current guidelines from the International Society of Ultrasound in Obstetrics and Gynecology do not recommend a systematic protocol for the prenatal identification of MCI [9].

There has been an ongoing debate concerning whether MCI should be regarded as a normal or abnormal variation of UCI and, if deemed abnormal, what the optimal strategies for monitoring and managing such pregnancies should be. A critical step toward establishing the universal prenatal identification of abnormal umbilical cord insertion or enabling targeted screening in high-risk pregnancies is the identification of potential risk factors for MCI. Previous studies have suggested various potential risk factors, including the use of assisted reproductive technology (ART), advanced maternal age, smoking, previous history of Cesarean section (CS), placenta previa, nulliparity, Caucasian ethnicity, and chronic hypertension [10,11,12,13,14,15].

This meta-analysis aimed to rigorously evaluate the current evidence regarding potential risk factors for MCI.

## 2. Materials and Methods

We planned this study and reported our results according to the Meta-Analysis Of Observational Studies in Epidemiology guidelines [16]. Our protocol was published in the International Prospective Register of Systematic Reviews under the number CRD42024546992. Patient consent and ethical approval were not required due to the nature of study, which was the synthesis of existing literature.

### 2.1. Search Strategy

We explored the research question, “Which population characteristics indicate an increased risk for MCI in singleton pregnancies?” using keywords like cord insertion, umbilical cord insertion, placental cord insertion, abnormal, aberrant, marginal, and battledore. The detailed search strategies are available in the Supplementary Materials. Searches were conducted in MEDLINE, Cochrane, and Scopus until 6 May 2024. The results were managed using Rayyan online software (https://new.rayyan.ai/). Additional studies were identified by examining references of relevant articles and performing supplementary searches online. After removing duplicates, the titles and abstracts were screened, followed by a thorough review of full texts for eligibility. The selection process was carried out blindly by two reviewers. Any disagreements were resolved by a third reviewer.

### 2.2. Selection Criteria

We considered observational cohort and case–control studies written in English that investigated risk factors in singleton pregnancies diagnosed with MCI, whether identified before or after delivery. The control group consisted of pregnancies with central or eccentric cord insertion (CCI). The exclusion criteria comprised multifetal pregnancies and pregnancies with aneuploidies or congenital anomalies. In instances where multiple studies utilized the same database covering overlapping time periods, we included only the study with the largest sample size to avoid duplication. Inclusion required that studies provide raw data on the investigated risk factors.

### 2.3. Data Extraction

A standardized template was developed for data extraction, capturing essential study characteristics, MCI definition, and timing of diagnosis, as well as the investigated risk factors and the relevant raw data. We contacted the authors to obtain missing data, when necessary.

### 2.4. Risk Factors of Interest

We analyzed risk factors reported by three or more studies, such as ART, maternal age, previous history of pregnancy termination, previous history of CS, preexisting diabetes, placenta previa, smoking, nulliparity, Caucasian race, chronic hypertension, and any other adequately reported risk factor, as per the protocol.

### 2.5. Quality and Bias Assessment

Two researchers independently assessed the quality of the included studies using the Newcastle–Ottawa scale, which evaluates selection, comparability, and outcome/risk factor ascertainment, assigning up to 9 points for the highest quality [17]. The Quality In Prognosis Studies (QUIPS) tool was also used to evaluate the risk of bias across six domains as low, moderate, or high risk of bias [18]. Discrepancies were resolved by a third reviewer.

### 2.6. Statistical Analysis

The original data were synthesized to calculate effect sizes and 95% confidence intervals (CIs) for various MCI risk factors using Review Manager 5.4 software. Relative risks (RRs) for binary outcomes were determined using the Mantel–Haenszel method, and mean differences (MDs) for continuous variables were calculated using the inverse variance method. Due to variability in observational studies, only a random-effects model was employed. Heterogeneity was assessed using the Cochran Q test (*p*-value threshold of 0.10) and I^2^ statistics [19]. Funnel plots were generated using the meta package [20] in R version 2.15.1 [21], and publication bias was assessed using Egger’s test with the dmetar package [22]. Publication bias was assessed for the risk factor with the largest included population.

### 2.7. Sensitivity Analysis

As per our protocol, a sensitivity analysis, including only prenatally identified cases, was conducted to improve the understanding of the prenatal diagnosis of MCI. Another sensitivity analysis excluded the studies with a high risk of bias (RoB), as determined by the QUIPS tool, aiming to verify the consistency of the results; for sensitivity analyses to be performed, at least three studies were needed.

## 3. Results

**Figure 1:**
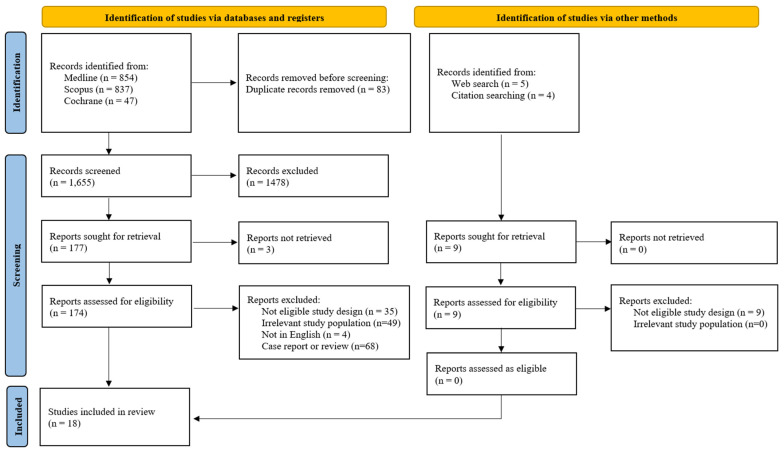
Study selection flow diagram.

We initially identified 1738 records through searches in the MEDLINE, Scopus, and Cochrane databases. An additional nine records were found via web searches and citation tracking. After eliminating 83 duplicate records, we screened 1655 unique records, from which we excluded 1478 for not meeting our criteria. This left us with 174 full-text articles to assess for eligibility, and 18 studies satisfied our inclusion criteria. The other methods yielded nine relevant articles, but none qualified for inclusion (Figure 1).

The selection process demonstrated a high level of agreement between the two reviewers, reflected by a Cohen’s kappa coefficient of 0.945. Consequently, we included a total of 14 cohort studies [3,4,10,11,12,13,14,15,23,24,25,26,27,28] and four case–control studies [29,30,31,32] in our final analysis. Details regarding the included studies are provided in Table 1.

### 3.1. Quality and Risk of Bias Assessment of the Studies

Newcastle–Ottawa Scale assessment revealed that four studies achieved excellent quality, with nine stars [11,23,24,28]. Five studies received eight stars [12,13,14,26,29] while seven earned seven stars [3,4,10,15,25,27,30], suggesting an adequate methodological approach. Finally, two studies scored five and four stars [31,32], depicting low study quality (Table 2). The most common weakness was found in the comparability category. A risk of bias visualization, according to QUIPS, was provided for every study next to each risk factor’s forest plot.

### 3.2. Raw Data Analysis

In total, we meta-analyzed 18 eligible studies. According to the data of 14 included cohort studies, which reported 51,463 cases of MCI and 901,020 cases of CCI, the prevalence of MCI in singleton pregnancies was calculated at 5.71%, with a 95% CI between 5.67% and 5.76%.

### 3.3. Risk Factor Analyses

**Figure 2:**
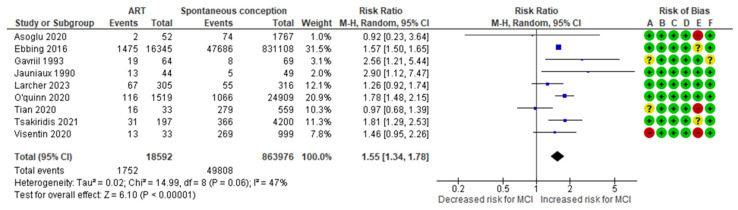
Forest plot investigating the association between ART and the risk of MCI in singleton pregnancies. Abbreviations: ART, assisted reproductive technology; CI, confidence interval; M–H, Mantel–Haenszel method; MCI, marginal cord insertion [10,11,12,13,14,15,29,30,32].

The ART-MCI meta-analysis combined data from six cohort [10,11,12,13,14,15] and three case–control [29,30,32] studies, evaluating a total of 1,752 MCI cases in the ART group, representing approximately 9.43% of the cases. In comparison, the spontaneous conception group recorded 49,808 MCI cases, representing about 5.76% of the control group. The analysis indicated a significantly higher incidence of MCI in the ART group compared to that in the spontaneous conception group, with an RR of 1.55 (95% CI 1.34 to 1.78). The included studies exhibited moderate heterogeneity (*p* = 0.06; I^2^ = 47%) (Figure 2).

**Figure 3:**
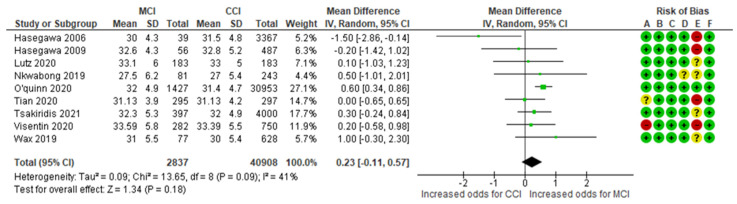
Forest plot investigating the association between maternal age and the risk of MCI in singleton pregnancies. Abbreviations: CCI, central/eccentric cord insertion; CI, confidence interval; IV, weighted mean difference; SD, standard deviation; MCI, marginal cord insertion [4,13,14,15,25,27,28].

The maternal age-MCI meta-analysis combined data from seven cohort [4,13,14,15,25,27,28] and two case–control [31,32] studies, evaluating a total of 2,837 MCI cases and 40,908 control cases of CCI. The analysis indicated a non-significant mean difference in maternal age between the MCI group and the control group, with an MD of 0.23 (95% CI −0.11 to +0.57). The included studies exhibited moderate heterogeneity (*p* = 0.09; I^2^ = 41%) (Figure 3).

**Figure 4:**
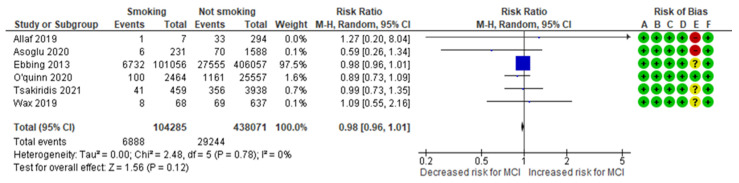
Forest plot investigating the association between smoking and the risk of MCI in singleton pregnancies. Abbreviations: CI, confidence interval; M–H, Mantel–Haenszel method; MCI, marginal cord insertion [3,10,13,14,23,28].

The smoking-MCI meta-analysis combined data from six cohort studies [3,10,13,14,23,28], evaluating a total of 6,888 MCI cases in the smoking group, representing approximately 6.60% of the cases. In comparison, 29,244 MCI cases were recorded in the non-smoking group, representing about 6.68%. The analysis indicated no significant difference in the incidence of the condition between smokers and non-smokers, with an RR of 0.98 (95% CI 0.96 to 1.01). The included studies did not exhibit heterogeneity (*p* = 0.78; I^2^ = 0%) (Figure 4).

**Figure 5:**
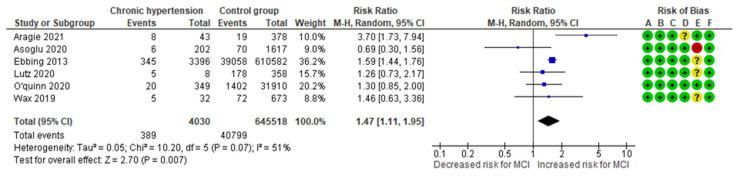
Forest plot investigating the association between chronic hypertension and the risk of MCI in singleton pregnancies. Abbreviations: CI, confidence interval; M–H, Mantel–Haenszel method; MCI, marginal cord insertion [10,13,23,26,27,28].

The chronic hypertension-MCI meta-analysis combined data from six cohort studies [10,13,23,26,27,28], evaluating a total of 389 MCI cases in the group of women with chronic hypertension, representing approximately 9.65% of the cases. In comparison, 40,799 MCI cases were recorded in the control group, representing about 6.32%. The analysis indicated a significantly higher incidence of MCI among women with chronic hypertension compared to that in the control group, with an RR of 1.47 (95% CI 1.11 to 1.95). The included studies exhibited moderate to substantial heterogeneity (*p* = 0.07; I^2^ = 51%) (Figure 5).

**Figure 6:**
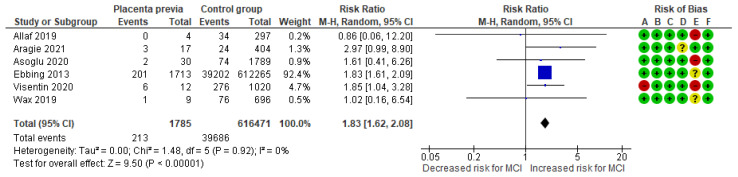
Forest plot investigating the association between placenta previa and the risk of MCI in singleton pregnancies. Abbreviations: CI, confidence interval; M–H, Mantel–Haenszel method; MCI, marginal cord insertion [3,10,15,23,26,28].

The placenta previa-MCI meta-analysis combined data from six cohort studies [3,10,15,23,26,28], evaluating a total of 213 MCI cases in the group complicated by placenta previa, representing approximately 11.93% of the cases. In comparison, 39,686 MCI cases were recorded in the control group, representing about 6.44%. The analysis indicated a significantly higher incidence of MCI in the placenta previa group compared to that in the control group, with an RR of 1.83 (95% CI 1.62 to 2.08). The included studies did not exhibit heterogeneity (*p* = 0.92; I^2^ = 0%) (Figure 6).

**Figure 7:**
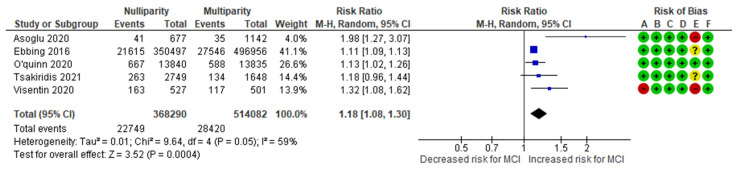
Forest plot investigating the association between nulliparity and the risk of MCI in singleton pregnancies. Abbreviations: CI, confidence interval; M–H, Mantel–Haenszel method; MCI, marginal cord insertion [10,11,13,14,15].

The nulliparity-MCI meta-analysis combined data from five cohort studies [10,11,13,14,15], evaluating a total of 22,749 MCI cases among nulliparous women, representing approximately 6.18% of the cases. In comparison, 28,420 MCI cases were recorded in the control group, representing about 5.53%. The analysis indicated a significantly higher incidence of MCI in the study group, with an RR of 1.18 (95% CI 1.08 to 1.30). The included studies exhibited moderate to substantial heterogeneity (*p* = 0.05; I^2^ = 59%) (Figure 7).

**Figure 8:**
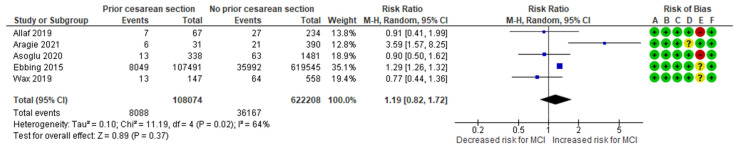
Forest plot investigating the association between prior Cesarean section and the risk of MCI in singleton pregnancies. Abbreviations: CI, confidence interval; M–H, Mantel–Haenszel method; MCI, marginal cord insertion [3,10,24,26,28].

The previous history of CS-MCI meta-analysis combined data from five cohort studies [3,10,24,26,28], evaluating a total of 8088 MCI cases among women with previous history CS, representing approximately 7.48% of the cases. In comparison, 36,167 MCI cases were recorded in the control group, representing about 5.81%. The analysis indicated no significant difference in the risk for MCI between the study group and the control group, with an RR of 1.19 (95% CI 0.82 to 1.72). The included studies exhibited high heterogeneity (*p* = 0.02; I^2^ = 64%) (Figure 8).

**Figure 9:**
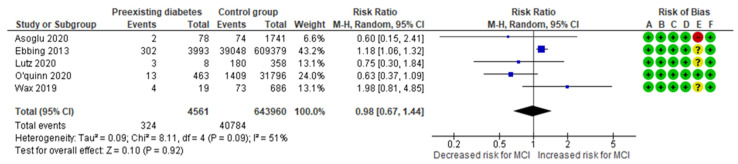
Forest plot investigating the association between preexisting diabetes and the risk of MCI in singleton pregnancies. Abbreviations: CI, confidence interval; M–H, Mantel–Haenszel method; MCI, marginal cord insertion [10,13,23,27,28].

The preexisting diabetes-MCI meta-analysis combined data from five cohort studies [10,13,23,27,28], evaluating a total of 324 MCI cases in the group of women with diagnosed preexisting diabetes, representing approximately 7.10% of the cases. In comparison, 40,784 MCI cases were recorded in the control group, representing about 6.33%. The analysis indicated no significant difference in the incidence of MCI between the study and the control group, with an RR of 0.98 (95% CI 0.67 to 1.44). The included studies exhibited moderate to substantial heterogeneity (*p* = 0.09; I^2^ = 51%) (Figure 9).

**Figure 10:**
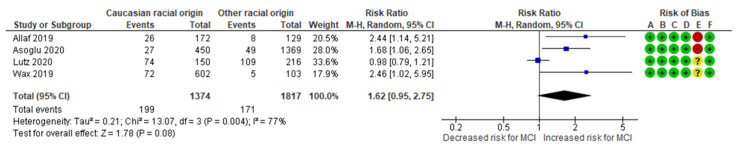
Forest plot investigating the association between Caucasian racial origin and the risk of MCI in singleton pregnancies. Abbreviations: CI, confidence interval; M–H, Mantel–Haenszel method; MCI, marginal cord insertion [3,10,27,28].

The Caucasian racial origin-MCI meta-analysis combined data from four cohort studies [3,10,27,28], evaluating a total of 199 MCI cases in the Caucasian group, representing approximately 14.48% of the cases. In comparison, 171 MCI cases were reported in the control group, representing about 9.41%. The analysis indicated no significant difference in the incidence of MCI between women of Caucasian racial origin and other racial origins, with an RR of 1.62 (95% CI 0.95 to 2.75). The included studies exhibited substantial heterogeneity (*p* = 0.004; I^2^ = 77%) (Figure 10).

The combined findings from our primary analysis are summarized and presented in Table 3.

### 3.4. Sensitivity Analyses Regarding Prenatal Diagnosis and Risk of Bias

Our sensitivity analysis was restricted to studies that provided data on the prenatal diagnosis of UCI. Out of the 18 studies reviewed, 9 included the prenatal identification of MCI. By excluding studies that confirmed MCI only postnatally, we were able to sufficiently evaluate all risk factors except for the Caucasian race risk factor. The sensitivity analysis’ findings aligned with those of the broader analysis, except for chronic hypertension and placenta previa, which lost their statistically significant associations but still showed increased RRs. Furthermore, while maternal age initially showed no association with MCI, a positive association emerged in the sensitivity analysis (Table 4 and Supplementary Figures S1–S8).

Moreover, we excluded studies identified with a high-risk of bias and re-evaluated the remaining data to ensure the results’ integrity and reliability. After this exclusion, seven out of eight risk factors showed results comparable to the initial findings (Table 4 and Supplementary Figures S9 and S11–S16). The only difference was in the association with maternal age, which changed from not significant in the initial analysis to a positive association in the risk of bias sensitivity analysis (Table 4 and Supplementary Figure S10).

### 3.5. Publication Bias

**Figure 11:**
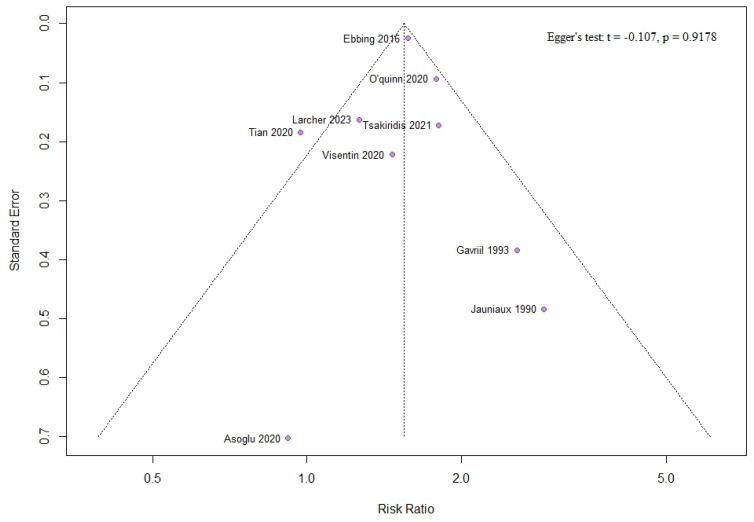
Funnel plot and Egger’s test for the use of assisted reproductive technology [10,11,12,13,14,15,29,30,32].

No evidence of publication bias was found for ART, as assessed by the funnel plot and Egger’s test (Figure 11).

## 4. Discussion

### 4.1. Main Findings

We found that the prevalence of MCI is 5.71%, while ART, chronic hypertension, placenta previa, and nulliparity were identified as significant risk factors for MCI among singleton pregnancies. Maternal age also emerged as a significant factor, if studies with high risk of bias were excluded.

### 4.2. Interpretation of the Findings

The ART-MCI analysis, which incorporated data from nine studies, constituted the largest population examined in our research. This analysis indicated that ART is associated with a 55% increased risk of MCI in singleton pregnancies, and this association remained significant, even when considering only cases with prenatally diagnosed MCI. The strength of these findings was further confirmed through a sensitivity analysis that excluded three studies identified as having a high risk of bias.

Our results are in line with those of a relevant meta-analysis that calculated an OR of 1.58 (95% CI 1.26–1.99); however, it is noteworthy that this previous meta-analysis included a broadly defined control group and also encompassed twin gestations [33]. Additionally, our findings are consistent with earlier epidemiological studies demonstrating that pregnancies achieved via ART are at increased risk for umbilico-placental abnormalities [34,35,36].

Although the exact mechanisms by which ART may disrupt placentation are not fully understood, several procedures associated with ART may introduce thermal, mechanical, and oxidative stresses. These stresses have the potential to alter the natural biological processes involved in reproduction [37]. Furthermore, since these interventions occur during critical periods of epigenetic reprogramming, placentation and pregnancy might be more susceptible to changes in DNA methylation [38].

The meta-analysis investigating the relationship between maternal age and MCI included nine studies and produced notable results. In our primary analysis, there was no statistically significant association (95% CI −0.11 to +0.57). However, both of our sensitivity analyses found statistically significant associations when limited to prenatally diagnosed cases (95% CI +0.21 to +0.70) and studies with low/medium risk of bias (95% CI +0.32 to +0.76). It appears that one study, which reported a negative association between maternal age and MCI, contributed to the higher heterogeneity observed in our main analysis, leading to the non-significant association due to the random-effects model used. When this study was excluded from the sensitivity analysis, the heterogeneity decreased, and statistical significance was achieved. Notably, ART, which is more common in women of advanced maternal age, could be a significant confounding factor.

Women with chronic hypertension were found to have an approximately 50% increased risk for MCI. This finding is particularly noteworthy, as most individual studies on this topic reported no significant association. Chronic hypertension is linked to altered placental shape [39], which results from the repeated branching of uteroplacental vascular trees, providing indirect support for the positive correlation between chronic hypertension and MCI identified in our study. However, the lack of consistent descriptions of chronic hypertension among the included studies—particularly concerning its severity, duration, and treatment—reduces the clinical value of this finding.

The diagnosis of placenta previa has been linked to a two-fold increase in the prevalence of MCI. Placenta previa is recognized as a well-established risk factor for both velamentous and marginal cord insertion. Despite this, most studies did not yield statistically significant results on their own [3,10,28,40].

An elevated risk for MCI, approximately 20% higher, was observed among nulliparous women. No pertinent studies elucidating the pathophysiological mechanisms were identified. We hypothesize that in multiparous women, physiological changes within the uterus enhance its receptivity to the placenta in subsequent pregnancies.

### 4.3. Strengths and Limitations

A key strength of our study is its comprehensive design, which allowed us to include a large number of studies and examine a wide range of potential risk factors. By applying strict inclusion criteria, we were able to obtain more reliable effect estimates and reduce variability among the studies analyzed. Our emphasis on enhancing the understanding of the prenatal diagnosis of MCI is further supported by our relevant sensitivity analysis, which enhances the generalizability of our findings.

However, this meta-analysis has several limitations. The included studies were observational, making them susceptible to inherent biases. In most studies, the MCI risk factors were not the primary outcome; they were only reported in the table of population characteristics, making them more susceptive to biases. Another limitation is the lack of systematic postnatal confirmation in studies where MCI was diagnosed prenatally. Moreover, the absence of adjusted effect measures in the included studies limited our ability to account for significant confounding variables.

## 5. Conclusions

This meta-analysis identified ART, chronic hypertension, placenta previa, and nulliparity as significant risk factors for marginal cord insertion. This knowledge may enable targeted screening for umbilical cord insertion anomalies in situations where universal screening is not routinely conducted. Additionally, our results highlight the need for further high-quality research that addresses potential confounding factors to confirm these associations. Understanding the underlying pathophysiological mechanisms of these relationships is essential to improve our knowledge and inform future obstetric practice.

## Figures and Tables

**Table 1:** Characteristics of the included studies.

First Author, Year	Country	Study Type	Study Period	Population MCI/CCI	Inclusion Criteria	Exclusion Criteria	Cord Insertion Site Diagnosis	Risk Factors
Allaf et al., 2019 [3]	USA	prospective cohort	2012–2015	34/267	singleton pregnancies with routine anatomical survey at 18–22 wk	chromosomal abnormalities, congenital malformations, VCI, loss to follow-up	antenatally, with 2D grayscale, color Doppler	smoking, placenta previa, prior CS, Caucasian race
Aragie et al., 2021 [26]	Ethiopia	prospective cohort	2020	27/383	singleton pregnancies	placenta specimens without intact cord, with pathology, bifurcated umbilical cord, and VCI	antenatally with US	chronic hypertension, placenta previa, prior CS
Asoglu et al., 2020 [10]	USA	retrospective cohort	2016–2018	76/1743	singleton pregnancies with at least one US scan at 16–32 wk	multiple pregnancies, VCI, pregnancy termination, deliveries < 24 wk, no US scan	antenatally with US at 16–32 wk by MFM specialist	ART, smoking, chronic hypertension, placenta previa, nulliparity, prior CS, preexisting diabetes, Caucasian race
Ebbing et al., 2013 [23]	Norway	retrospective cohort	1999–2009	39,403/574,575	singleton pregnancies delivered at 16–45 wk	multiple pregnancies	postnatally (form completed by the attending midwife or physician shortly after delivery)	smoking, chronic hypertension, placenta previa, preexisting diabetes
Ebbing et al., 2015 [24]	Norway	retrospective cohort	1999–2011	44,041/727,995	singleton pregnancies delivered at 16–45 wk	multiple pregnancies	postnatally (inspection of cord insertion during the delivery)	prior CS
Ebbing et al., 2016 [11]	Norway	retrospective cohort	1999–2013	54,693/751,079	singleton pregnancies delivered at 16–45 wk	multiple pregnancies	postnatally (inspection of cord insertion during the delivery)	ART, nulliparity
Gavriil et al., 1993 [30]	Belgium	retrospective case–control	not reported	27/106	ART and intrauterine embryo transfer pregnancies	includes multiple pregnancies but reports separate raw data for singleton pregnancies	postnatally through pathologic examination	ART
Hasegawa et al., 2006 [4]	Japan	retrospective cohort	2002–2004	39/3367	singleton pregnancies	women who first visited the hospital after 20 wk	postnatally (inspection of cord insertion during the delivery)	maternal age
Hasegawa et al., 2009 [25]	Japan	retrospective cohort	2005–2006	56/487	singleton pregnancies delivered at 22–41 wk	multiple pregnancies	postnatally (inspection of cord insertion during the delivery)	maternal age
Jauniaux et al., 1990 [29]	Belgium	retrospective case–control	1985–1988	18/75	singleton pregnancies	multiple pregnancies, pregnancies with early vanishing twin phenomenon	postnatal macroscopic examination	ART
Larcher et al., 2023 [12]	Italy	prospective cohort	2019–2021	122/499	singleton pregnancies	prior CS, unmatched pregnancies, delivery < 32 wk	antenatal US scan at 11–14, 19–22, and 32–35 wk	ART
Lutz et al., 2020 [27]	USA	retrospective cohort	2014–2016	183/183	singleton pregnancies	multiple pregnancies, chromosomal abnormalities, maternal comorbidities, congenital anomalies, VCI, placenta previa, loss to follow-up, delivery outside institution	Antenatally by routine fetal survey US by MFM specialist	maternal age, chronic hypertension, preexisting diabetes, Caucasian race
Nkwabong et al., 2019 [31]	Cameroon	retrospective case–control	2017–2018	81/243	singleton pregnancies	multiple pregnancies, undated pregnancies	postnatal inspection of cord insertion	maternal age
O’Quinn et al., 2020 [13]	Canada	retrospective cohort	2012–2015	1427/30,953	singleton pregnancies with completed anatomic survey, delivered > 24^+6^ wk	multiple pregnancies, placenta previa, vasa previa, no documented cord insertion type, fetal anomalies	antenatally (at the 18–21 wk with US)	ART, maternal age, smoking, chronic hypertension, nulliparity, preexisting diabetes
Tian et al., 2020 [32]	China	retrospective case–control	2013–2017	295/297	singleton pregnancies free of complications such as diabetes, hypertension, or anemia	multiple pregnancies	antenatal US at 23–25 wk, by five qualified sonographers	ART, maternal age
Tsakiridis et al., 2021 [14]	Greece	retrospective cohort	2016–2020	397/4000	singleton pregnancies with second trimester anomaly US at 20^+0^–23^+6^ wk	fetal abnormalities, vasa previa, single umbilical artery	antenatally (anomaly US at 20^+0^–23^+6^ wk)	ART, maternal age, smoking, nulliparity
Visentin et al., 2021 [15]	Italy	retrospective cohort	2016–2017	282/750	singleton pregnancies delivered > 24 wk	multiple pregnancies, miscarriages, voluntary abortions	postnatally (according to placenta pathology records)	ART, maternal age, placenta previa, nulliparity
Wax et al., 2019 [28]	USA	retrospective cohort	2012–2016	77/627	nonanomalous singleton fetus with US scan at 18 to 21^+6^ wk	pregnancies with VCI, delivered at an outside institution	antenatally, transabdominal grayscale imaging and color Doppler US, images reviewed by MFM specialist	maternal age, smoking, chronic hypertension, placenta previa, female sex, prior CS, preexisting diabetes, Caucasian race

Abbreviations: ART, assisted reproductive technology; CCI, central/eccentric cord insertion; CS, Cesarean section; MCI, marginal cord insertion; MFM, maternal fetal medicine; US, ultrasound; VCI, velamentous cord insertion; wk, gestational week.

**Table 2:** Quality assessment of the included studies according to the Newcastle–Ottawa Scale.

First Author, Year	Study Type	S1	S2	S3	S4	C	O1	O2	O3	Total
Allaf et al., 2019 [3]	retrospective cohort	a *	a *	a *	a *	-	b *	a *	a *	7
Aragie et al., 2021 [26]	prospective cohort	a *	a *	a *	a *	a *,b *	c	a *	a *	8
Asoglu et al., 2020 [10]	retrospective cohort	b *	a *	a *	a *	-	b *	a *	a *	7
Ebbing et al., 2013 [23]	retrospective cohort	a *	a *	a *	a *	a *,b *	b *	a *	a *	9
Ebbing et al., 2015 [24]	retrospective cohort	a *	a *	a *	a *	a *,b *	b *	a *	a *	9
Ebbing et al., 2016 [11]	retrospective cohort	a *	a *	a *	a *	a *,b *	b *	a *	a *	9
Gavriil et al., 1993 [30]	retrospective case–control	a *	b	a *	a *	a *	b *	a *	a *	7
Hasegawa et al., 2006 [4]	retrospective cohort	b *	a *	a *	a *	-	b *	a *	a *	7
Hasegawa et al., 2009 [25]	retrospective cohort	b *	a *	a *	a *	-	b *	a *	a *	7
Jauniaux et al., 1990 [29]	retrospective case–control	b *	a *	a *	a *	a *	a *	a *	a *	8
Larcher et al., 2023 [12]	prospective cohort	b *	a *	a *	a *	a *	b *	a *	a *	8
Lutz et al., 2020 [27]	retrospective cohort	b *	a *	a *	a *	-	b *	a *	a *	7
Nkwabong et al., 2019 [31]	retrospective case–control	b	a *	a *	a *	-	d	a *	a *	5
O’Quinn et al., 2020 [13]	retrospective cohort	a *	a *	a *	a *	b *	b *	a *	a *	8
Tian et al., 2020 [32]	retrospective case–control	b	b	c	a *	-	b *	a *	a *	4
Tsakiridis et al., 2021 [14]	retrospective cohort	b *	a *	a *	a *	a *	b *	a *	a *	8
Visentin et al., 2021 [15]	retrospective cohort	c	a *	a *	a *	a *	b *	a *	a *	7
Wax et al., 2019 [28]	retrospective cohort	a *	a *	a *	a *	a *,b *	a *	a *	a *	9

Abbreviations: a, first answer according to Newcastle–Ottawa Scale; b, second answer according to Newcastle–Ottawa Scale; c, third answer according to Newcastle–Ottawa Scale; S, selection; C, comparability; O, outcome; *, attribution of a star according to NOS.

**Table 3:** Results of the meta-analysis regarding risk factors for marginal cord insertion in singleton pregnancies.

Risk Factor	Studies	MCI Cases/Total Cases (Exposed to Risk Factor)	MCI Cases/Total Cases (Not Exposed to Risk Factor)	RR	95% CI	I^2^; *p*-Value
Assisted reproductive technology	9	1752/18,592 (9.43%)	49,808/863,976 (5.76%)	1.55	1.34–1.78	47%; 0.06
Maternal age	9	2837 (MCI cases)	40,908 (CCI cases)	+0.23 (MD)	−0.11 to +0.57	41%; 0.09
Smoking	6	6888/104,285 (6.60%)	29,244/438,071 (6.68%)	0.98	0.96–1.01	0%; 0.78
Chronic hypertension	6	389/4030 (9.65%)	40,799/645,518 (6.32%)	1.47	1.11–1.95	51%; 0.07
Placenta previa	6	213/1785 (11.93%)	39,686/616,471 (6.44%)	1.83	1.62–2.08	0%; 0.92
Nulliparity	5	22,749/368,290 (6.18%)	28,420/514,082 (5.53%)	1.18	1.08–1.30	59%; 0.05
Prior Cesarean section	5	8088/108,074 (7.48%)	36,167/622,208 (5.81%)	1.19	0.82–1.72	64%; 0.02
Preexisting diabetes	5	324/4561 (7.10%)	40,784/643,960 (6.33%)	0.98	0.67–1.44	51%; 0.09
Caucasian race	4	199/1374 (14.48%)	171/1817 (9.41%)	1.62	0.95–2.75	77%; 0.004

Abbreviations: CCI, central/eccentric cord insertion; CI, confidence interval; I^2^ (heterogeneity in meta-analysis); *p*-value, Cochran Q test’s *p*-value; RR, relative risk; MCI, marginal cord insertion.

**Table 4:** Confidence intervals from all the analyses performed.

Risk Factor	Overall Analysis		Prenatally Diagnosed		RoB Sensitivity Analysis	
	RR	95% CI	RR	95% CI	RR	95% CI
Assisted reproductive technology	1.55	1.34–1.78	1.42	1.08–1.85	1.64	1.46–1.84
Maternal age	+0.23 (MD)	−0.11 to +0.57	+0.46 (MD)	0.21 to 0.70	+0.54 (MD)	0.32 to 0.76
Smoking	0.98	0.96–1.01	0.92	0.78–1.07	0.98	0.96–1.01
Chronic hypertension	1.47	1.11–1.95	1.42	0.91–2.23	1.58	1.24–2.00
Placenta previa	1.83	1.62–2.08	1.89	0.90–3.98	1.84	1.62–2.09
Nulliparity	1.18	1.08–1.30	1.26	1.02–1.55	1.11	1.09–1.13
Prior Cesarean section	1.19	0.82–1.72	1.17	0.63–2.20	1.40	0.78–2.51
Preexisting diabetes	0.98	0.67–1.44	0.86	0.49-1.50	1.01	0.67–1.54
Caucasian race	1.62	0.95–2.75				

Abbreviations: CI, confidence interval; RR, relative risk; RoB, risk of bias.

## Supplementary Materials

The following supporting information can be downloaded at: https://www.mdpi.com/article/10.3390/jcm13237438/s1, Figure S1. Forest plot demonstrating the risk for prenatally diagnosed MCI in singleton pregnancies relative to the use of ART. Figure S2. Forest plot demonstrating the risk for prenatally diagnosed MCI in singleton pregnancies relative to mean maternal age. Figure S3. Forest plot demonstrating the risk for prenatally diagnosed MCI in singleton pregnancies relative to smoking. Figure S4. Forest plot demonstrating the risk for prenatally diagnosed MCI in singleton pregnancies relative to chronic hypertension. Figure S5. Forest plot demonstrating the risk for prenatally diagnosed MCI in singleton pregnancies relative to placenta previa. Figure S6. Forest plot demonstrating the risk for prenatally diagnosed MCI in singleton pregnancies relative to nulliparity. Figure S7. Forest plot demonstrating the risk for prenatally diagnosed MCI in singleton pregnancies relative to history of prior cesarean section. Figure S8. Forest plot demonstrating the risk for prenatally diagnosed MCI in singleton pregnancies relative to preexisting diabetes. Figure S9. Risk of bias sensitivity analysis regarding ART—VCI association in singleton pregnancies. Figure 10. Risk of bias sensitivity analysis regarding mean maternal age—VCI association in singleton pregnancies. Figure S11. Risk of bias sensitivity analysis regarding smoking—VCI association in singleton pregnancies. Figure S12. Risk of bias sensitivity analysis regarding chronic hypertension—VCI association in singleton pregnancies. Figure S13. Risk of bias sensitivity analysis regarding placenta previa—VCI association in singleton pregnancies. Figure S14. Risk of bias sensitivity analysis regarding nulliparity—VCI association in singleton pregnancies. Figure S15. Risk of bias sensitivity analysis regarding history of prior cesarean section—VCI association in singleton pregnancies. Figure S16. Risk of bias sensitivity analysis regarding preexisting diabetes—VCI association in singleton pregnancies.
