# Supplementary material for: Risk Factors of Marginal Cord Insertion in Singleton Pregnancies: A Systematic Review and Meta-Analysis
Source: J Clin Med. 2024 Dec 6;13(23):7438. doi: 10.3390/jcm13237438 (PMC11642487; doi:10.3390/jcm13237438)
Supplement: Supplementary file 1 [file jcm-13-07438-s001.zip › jcm-3295088-supplementary.pdf]

Figure S1. Forest plot demonstrating the risk for prenatally diagnosed MCI in singleton pregnancies relative to the use of ART.

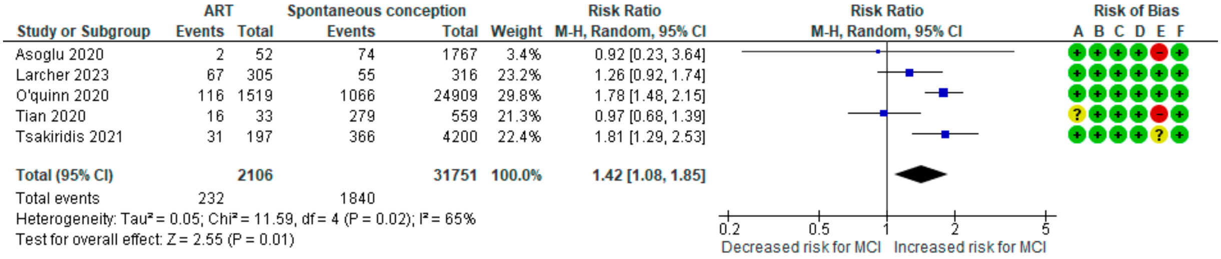

Abbreviations: ART, assisted reproductive technology; CI, confidence interval; M-H, Mantel–Haenszel method; MCI, marginal cord insertion

Figure S2. Forest plot demonstrating the risk for prenatally diagnosed MCI in singleton pregnancies relative to mean maternal age.

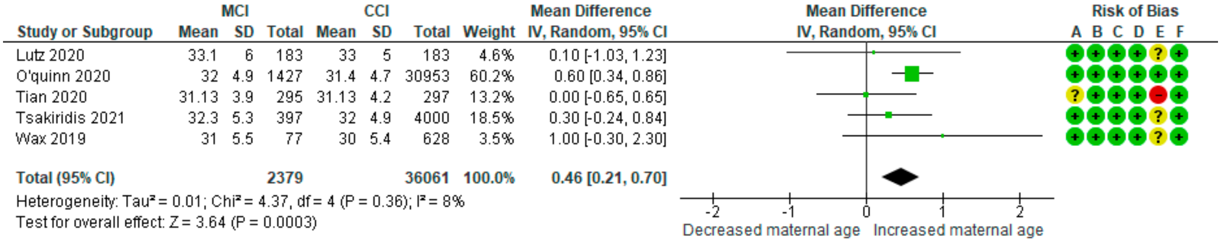

Abbreviations: CCI, central/eccentric cord insertion; CI, confidence interval; IV, weighted mean difference; SD, standard deviation; MCI, marginal cord insertion

Figure S3. Forest plot demonstrating the risk for prenatally diagnosed MCI in singleton pregnancies relative to smoking.

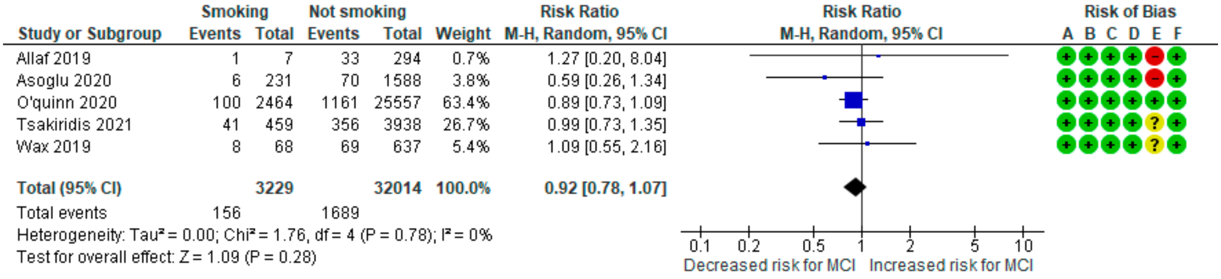

Abbreviations: CI, confidence interval; M-H, Mantel–Haenszel method; MCI, marginal cord insertion

Figure S4. Forest plot demonstrating the risk for prenatally diagnosed MCI in singleton pregnancies relative to chronic hypertension.

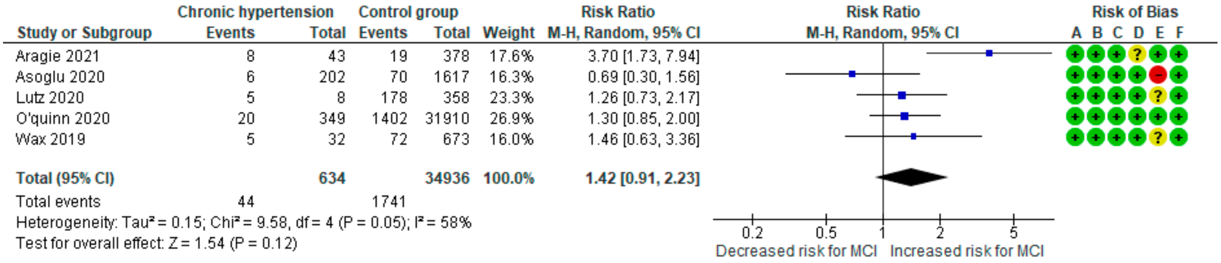

Abbreviations: CI, confidence interval; M-H, Mantel–Haenszel method; MCI, marginal cord insertion

Figure S5. Forest plot demonstrating the risk for prenatally diagnosed MCI in singleton pregnancies relative to placenta previa.

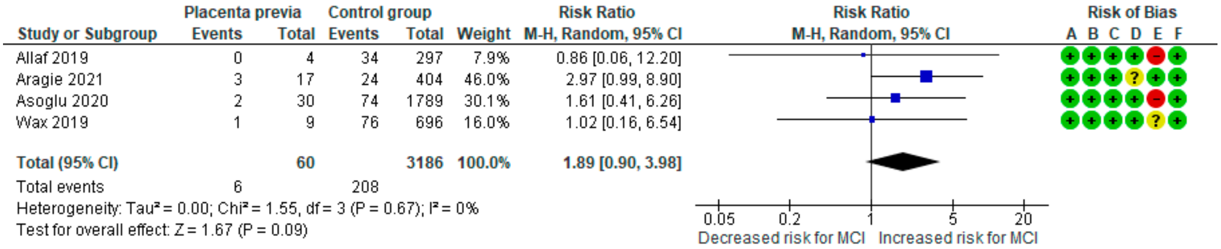

Abbreviations: CI, confidence interval; M-H, Mantel–Haenszel method; MCI, marginal cord insertion

Figure S6. Forest plot demonstrating the risk for prenatally diagnosed MCI in singleton pregnancies relative to nulliparity.

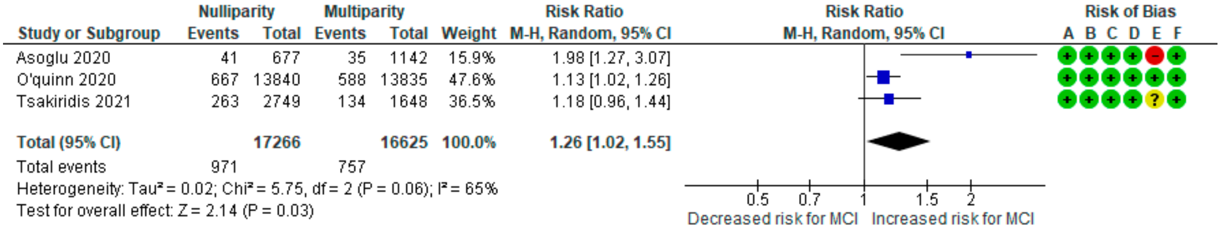

Abbreviations: CI, confidence interval; M-H, Mantel–Haenszel method; MCI, marginal cord insertion

Figure S7. Forest plot demonstrating the risk for prenatally diagnosed MCI in singleton pregnancies relative to history of prior cesarean section.

Study or Subgroup	Prior cesarean section Events	Prior cesarean section Total	No prior cesarean section Events	No prior cesarean section Total	Weight	Risk Ratio M-H, Random, 95% CI
Allaf 2019	7	67	27	234	22.9%	0.91 [0.41, 1.99]
Aragie 2021	6	31	21	390	22.0%	3.59 [1.57, 8.25]
Asoglu 2020	13	338	63	1481	27.3%	0.90 [0.50, 1.62]
Wax 2019	13	147	64	558	27.7%	0.77 [0.44, 1.36]
**Total (95% CI)**		**583**		**2663**	**100.0%**	**1.17 [0.63, 2.20]**
Total events	39		175			

Heterogeneity: Tau² = 0.29; Chi² = 10.22, df = 3 (P = 0.02); I² = 71%
Test for overall effect: Z = 0.50 (P = 0.62)

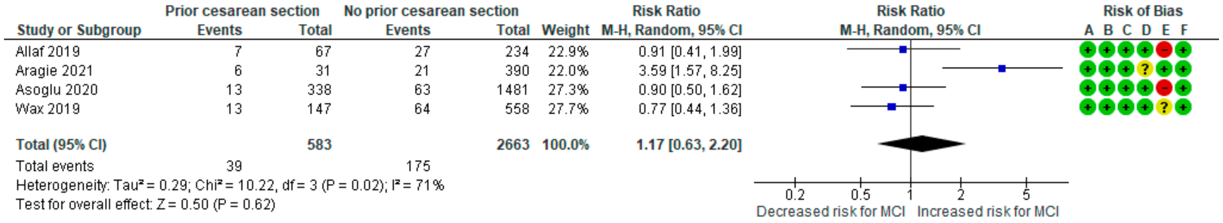

Abbreviations: CI, confidence interval; M-H, Mantel−Haenszel method; MCI, marginal cord insertion

Figure S8. Forest plot demonstrating the risk for prenatally diagnosed MCI in singleton pregnancies relative to preexisting diabetes.

Study or Subgroup	Preexisting diabetes Events	Preexisting diabetes Total	Control group Events	Control group Total	Weight	Risk Ratio M-H, Random, 95% CI
Asoglu 2020	2	78	74	1741	12.9%	0.60 [0.15, 2.41]
Lutz 2020	3	8	180	358	23.8%	0.75 [0.30, 1.84]
O'quinn 2020	13	463	1409	31796	39.5%	0.63 [0.37, 1.09]
Wax 2019	4	19	73	686	23.9%	1.98 [0.81, 4.85]
**Total (95% CI)**		**568**		**34581**	**100.0%**	**0.86 [0.49, 1.50]**
Total events	22		1736			

Heterogeneity: Tau² = 0.13; Chi² = 5.04, df = 3 (P = 0.17); I² = 41%
Test for overall effect: Z = 0.53 (P = 0.59)

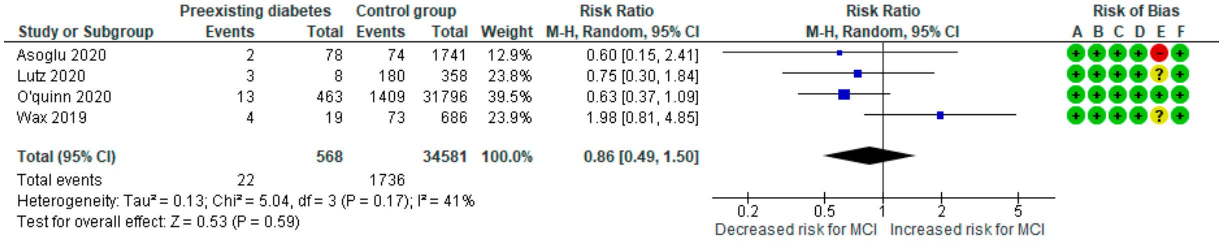

Abbreviations: CI, confidence interval; M-H, Mantel−Haenszel method; MCI, marginal cord insertion

Figure S9. Risk of bias sensitivity analysis regarding ART - VCI association in singleton pregnancies.

Study or Subgroup	ART Events	ART Total	Natural conception Events	Natural conception Total	Weight	Risk Ratio M-H, Random, 95% CI
Asoglu 2020	2	52	74	1767		Not estimable
Ebbing 2016	1475	16345	47686	831108	51.4%	1.57 [1.50, 1.65]
Gavriil 1993	19	64	8	69	2.4%	2.56 [1.21, 5.44]
Jauniaux 1990	13	44	5	49	1.5%	2.90 [1.12, 7.47]
Larcher 2023	67	305	55	316	11.0%	1.26 [0.92, 1.74]
O'quinn 2020	116	1519	1066	24909	23.7%	1.78 [1.48, 2.15]
Tian 2020	16	33	279	559		Not estimable
Tsakiridis 2021	31	197	366	4200	10.0%	1.81 [1.29, 2.53]
Visentin 2020	13	33	269	999		Not estimable
**Total (95% CI)**		**18474**		**860651**	**100.0%**	**1.64 [1.46, 1.84]**
Total events	1721		49186			

Heterogeneity: Tau² = 0.01; Chi² = 7.31, df = 5 (P = 0.20); I² = 32%
Test for overall effect: Z = 8.20 (P < 0.00001)

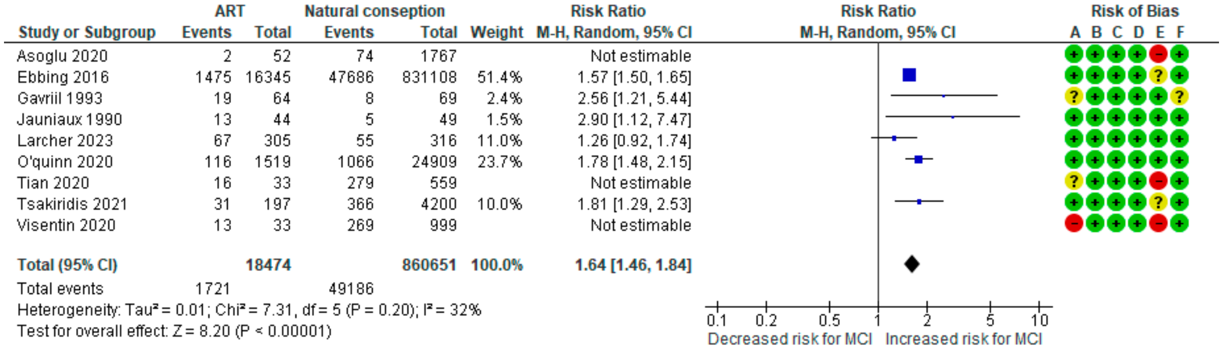

Abbreviations: ART, assisted reproductive technology; CI, confidence interval; M-H, Mantel−Haenszel method; MCI, marginal cord insertion

Figure 10. Risk of bias sensitivity analysis regarding mean maternal age - VCI association in singleton pregnancies.

Study or Subgroup	MCI Mean	MCI SD	MCI Total	CCI Mean	CCI SD	CCI Total	Weight	Mean Difference IV, Random, 95% CI
Hasegawa 2006	30	4.3	39	31.5	4.8	3367		Not estimable
Hasegawa 2009	32.6	4.3	56	32.8	5.2	487		Not estimable
Lutz 2020	33.1	6	183	33	5	183	3.9%	0.10 [-1.03, 1.23]
Nkwabong 2019	27.5	6.2	81	27	5.4	243	2.2%	0.50 [-1.01, 2.01]
O'quinn 2020	32	4.9	1427	31.4	4.7	30953	74.0%	0.60 [0.34, 0.86]
Tian 2020	31.13	3.9	295	31.13	4.2	297		Not estimable
Tsakiridis 2021	32.3	5.3	397	32	4.9	4000	16.9%	0.30 [-0.24, 0.84]
Visentin 2020	33.59	5.8	282	33.39	5.5	750		Not estimable
Wax 2019	31	5.5	77	30	5.4	628	3.0%	1.00 [-0.30, 2.30]
**Total (95% CI)**			**2165**			**36007**	**100.0%**	**0.54 [0.32, 0.76]**

Heterogeneity: Tau² = 0.00; Chi² = 2.02, df = 4 (P = 0.73); I² = 0%
Test for overall effect: Z = 4.73 (P < 0.00001)

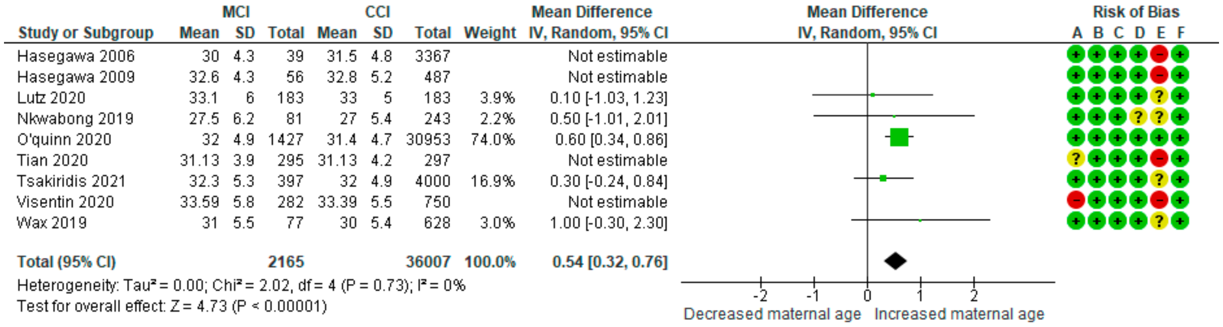

Abbreviations: CCI, central/eccentric cord insertion; CI, confidence interval; IV, weighted mean difference; SD, standard deviation; MCI, marginal cord insertion

Figure S11. Risk of bias sensitivity analysis regarding smoking - VCI association in singleton pregnancies.

Study or Subgroup	Smoking Events	Smoking Total	Not smoking Events	Not smoking Total	Weight	Risk Ratio M-H, Random, 95% CI
Allaf 2019	1	7	33	294		Not estimable
Asoglu 2020	6	231	70	1588		Not estimable
Ebbing 2013	6732	101056	27555	406057	97.6%	0.98 [0.96, 1.01]
O'quinn 2020	100	2464	1161	25557	1.6%	0.89 [0.73, 1.09]
Tsakiridis 2021	41	459	356	3938	0.7%	0.99 [0.73, 1.35]
Wax 2019	8	68	69	637	0.1%	1.09 [0.55, 2.16]
**Total (95% CI)**		**104047**		**436189**	**100.0%**	**0.98 [0.96, 1.01]**
Total events	6881		29141			

Heterogeneity: Tau² = 0.00; Chi² = 0.93, df = 3 (P = 0.82); I² = 0%
Test for overall effect: Z = 1.53 (P = 0.13)

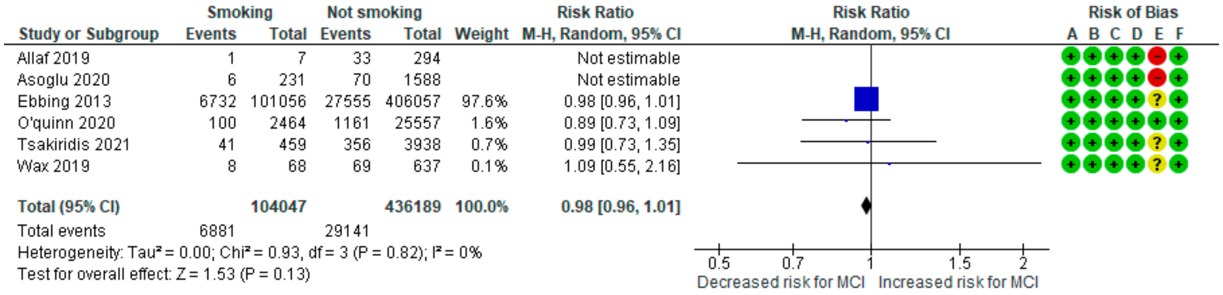

Abbreviations: CI, confidence interval; M-H, Mantel–Haenszel method; MCI, marginal cord insertion

Figure S12. Risk of bias sensitivity analysis regarding chronic hypertension - VCI association in singleton pregnancies.

Study or Subgroup	Chronic hypertension Events	Total	Control group Events	Total	Weight	Risk Ratio M-H, Random, 95% CI
Aragie 2021	8	43	19	378	8.3%	3.70 [1.73, 7.94]
Asoglu 2020	6	202	70	1617		Not estimable
Ebbing 2013	345	3396	39058	610582	50.6%	1.59 [1.44, 1.76]
Lutz 2020	5	8	178	358	14.1%	1.26 [0.73, 2.17]
O'quinn 2020	20	349	1402	31910	19.9%	1.30 [0.85, 2.00]
Wax 2019	5	32	72	673	7.1%	1.46 [0.63, 3.36]
**Total (95% CI)**		**3828**		**643901**	**100.0%**	**1.58 [1.24, 2.00]**
Total events	383		40729			

Heterogeneity: Tau² = 0.03; Chi² = 6.26, df = 4 (P = 0.18); I² = 36%
Test for overall effect: Z = 3.74 (P = 0.0002)

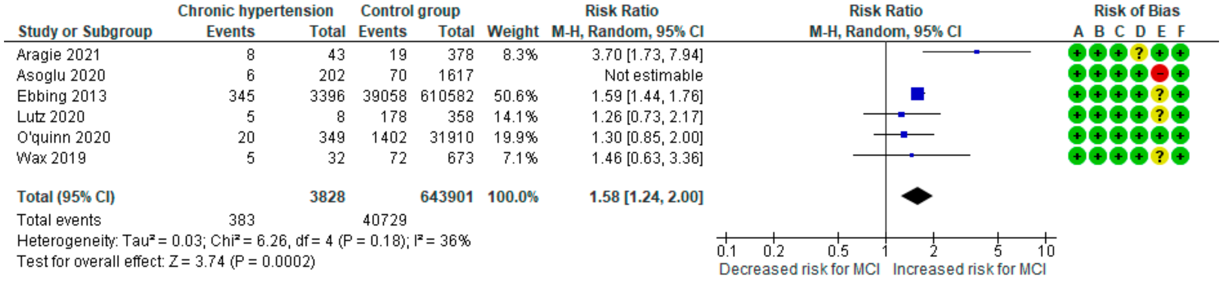

Abbreviations: CI, confidence interval; M-H, Mantel–Haenszel method; MCI, marginal cord insertion

Figure S13. Risk of bias sensitivity analysis regarding placenta previa - VCI association in singleton pregnancies.

Study or Subgroup	Placenta previa Events	Total	Control group Events	Total	Weight	Risk Ratio M-H, Random, 95% CI
Allaf 2019	0	4	34	297		Not estimable
Aragie 2021	3	17	24	404	1.4%	2.97 [0.99, 8.90]
Asoglu 2020	2	30	74	1789		Not estimable
Ebbing 2013	201	1713	39202	612265	98.1%	1.83 [1.61, 2.09]
Visentin 2020	6	12	276	1020		Not estimable
Wax 2019	1	9	76	696	0.5%	1.02 [0.16, 6.54]
**Total (95% CI)**		**1739**		**613365**	**100.0%**	**1.84 [1.62, 2.09]**
Total events	205		39302			

Heterogeneity: Tau² = 0.00; Chi² = 1.13, df = 2 (P = 0.57); I² = 0%
Test for overall effect: Z = 9.26 (P < 0.00001)

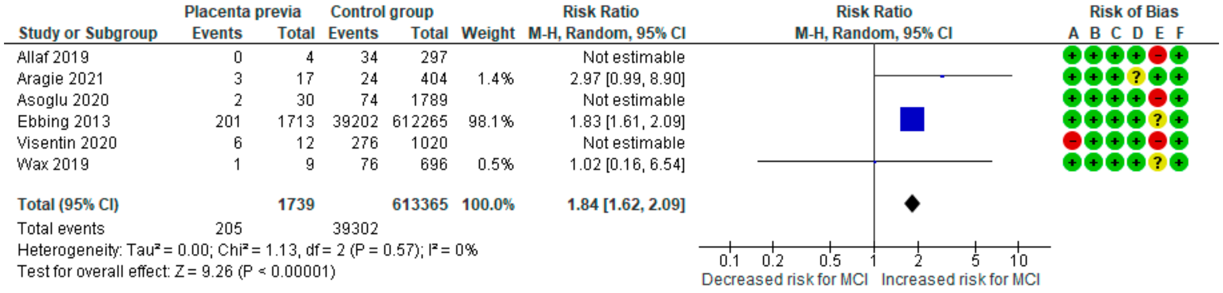

Abbreviations: CI, confidence interval; M-H, Mantel–Haenszel method; MCI, marginal cord insertion

Figure S14. Risk of bias sensitivity analysis regarding nulliparity - VCI association in singleton pregnancies.

Study or Subgroup	Nulliparity Events	Total	Multiparity Events	Total	Weight	Risk Ratio M-H, Random, 95% CI
Asoglu 2020	41	677	35	1142		Not estimable
Ebbing 2016	21615	350497	27546	496956	96.8%	1.11 [1.09, 1.13]
O'quinn 2020	667	13840	588	13835	2.5%	1.13 [1.02, 1.26]
Tsakiridis 2021	263	2749	134	1648	0.7%	1.18 [0.96, 1.44]
Visentin 2020	163	527	117	501		Not estimable
**Total (95% CI)**		**367086**		**512439**	**100.0%**	**1.11 [1.09, 1.13]**
Total events	22545		28268			

Heterogeneity: Tau² = 0.00; Chi² = 0.41, df = 2 (P = 0.81); I² = 0%
Test for overall effect: Z = 12.40 (P < 0.00001)

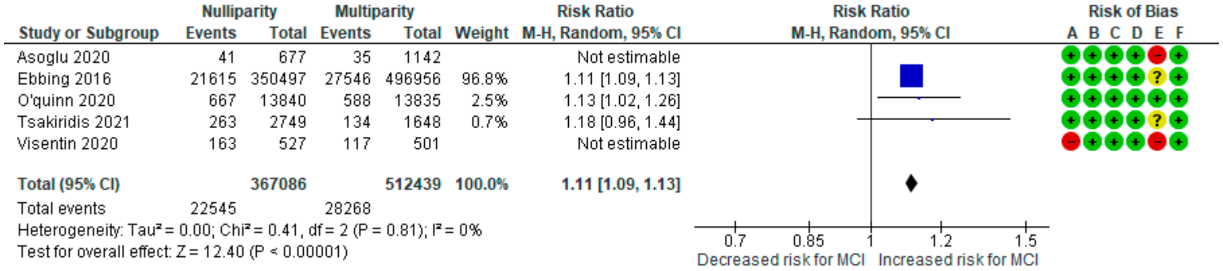

Abbreviations: CI, confidence interval; M-H, Mantel–Haenszel method; MCI, marginal cord insertion

Figure S15. Risk of bias sensitivity analysis regarding history of prior cesarean section - VCI association in singleton pregnancies.

Study or Subgroup	Prior cesarean section Events	Prior cesarean section Total	No prior cesarean section Events	No prior cesarean section Total	Weight	Risk Ratio M-H, Random, 95% CI
Allaf 2019	7	67	27	234		Not estimable
Aragie 2021	6	31	21	390	23.7%	3.59 [1.57, 8.25]
Asoglu 2020	13	338	63	1481		Not estimable
Ebbing 2015	8049	107491	35992	619545	44.8%	1.29 [1.26, 1.32]
Wax 2019	13	147	64	558	31.6%	0.77 [0.44, 1.36]
**Total (95% CI)**		**107669**		**620493**	**100.0%**	**1.40 [0.78, 2.51]**
Total events	8068		36077			

Heterogeneity: Tau² = 0.20; Chi² = 9.01, df = 2 (P = 0.01); I² = 78%
Test for overall effect: Z = 1.11 (P = 0.27)

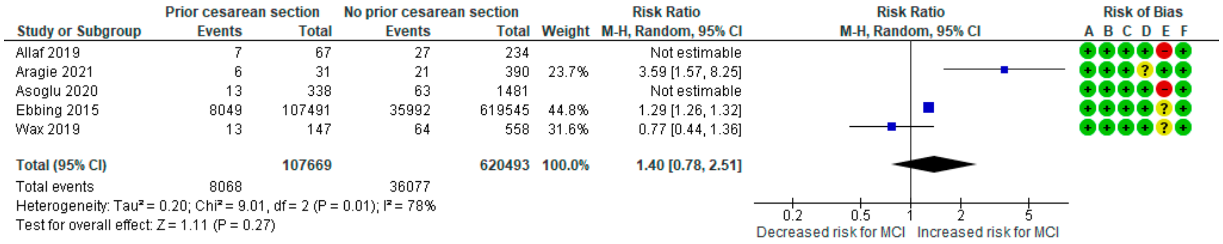

Abbreviations: CI, confidence interval; M-H, Mantel–Haenszel method; MCI, marginal cord insertion

Figure S16. Risk of bias sensitivity analysis regarding preexisting diabetes - VCI association in singleton pregnancies.

Study or Subgroup	Preexisting diabetes Events	Preexisting diabetes Total	Control group Events	Control group Total	Weight	Risk Ratio M-H, Random, 95% CI
Asoglu 2020	2	78	74	1741		Not estimable
Ebbing 2013	302	3993	39048	609379	44.6%	1.18 [1.06, 1.32]
Lutz 2020	3	8	180	358	14.6%	0.75 [0.30, 1.84]
O'quinn 2020	13	463	1409	31796	26.0%	0.63 [0.37, 1.09]
Wax 2019	4	19	73	686	14.7%	1.98 [0.81, 4.85]
**Total (95% CI)**		**4483**		**642219**	**100.0%**	**1.01 [0.67, 1.54]**
Total events	322		40710			

Heterogeneity: Tau² = 0.10; Chi² = 7.26, df = 3 (P = 0.06); I² = 59%
Test for overall effect: Z = 0.06 (P = 0.95)

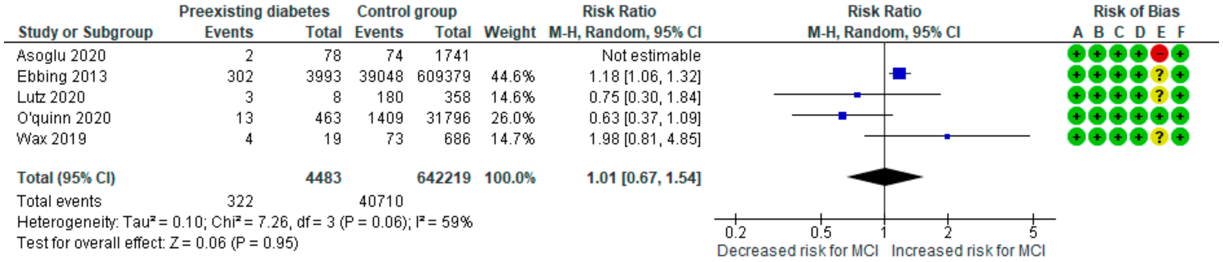

Abbreviations: CI, confidence interval; M-H, Mantel–Haenszel method; MCI, marginal cord insertion
